# Ethnicity Modifies Associations between Cardiovascular Risk Factors and Disease Severity in Parallel Dutch and Singapore Coronary Cohorts

**DOI:** 10.1371/journal.pone.0132278

**Published:** 2015-07-06

**Authors:** Crystel M. Gijsberts, Aruni Seneviratna, Leonardo P. de Carvalho, Hester M. den Ruijter, Puwalani Vidanapthirana, Vitaly Sorokin, Pieter Stella, Pierfrancesco Agostoni, Folkert W. Asselbergs, A. Mark Richards, Adrian F. Low, Chi-Hang Lee, Huay Cheem Tan, Imo E. Hoefer, Gerard Pasterkamp, Dominique P. V. de Kleijn, Mark Y. Chan

**Affiliations:** 1 Laboratory of Experimental Cardiology, University Medical Center Utrecht, Utrecht, The Netherlands; 2 The Netherlands Heart Institute (ICIN), Utrecht, The Netherlands; 3 Cardiac Department, National University Heart Centre, National University Hospital, Singapore, Singapore; 4 Department of Surgery, Yong Loo Lin School of Medicine, National University of Singapore, Singapore, Singapore; 5 Cardiology Department, University Medical Center Utrecht, Utrecht, The Netherlands; 6 Durrer Center for Cardiogenetic Research, ICIN-Netherlands Heart Institute, Utrecht, The Netherlands; 7 Institute of Cardiovascular Science, faculty of Population Health Sciences, University College London, London, United Kingdom; 8 Cardiovascular Research Institute (CVRI), National University Heart Centre (NUHCS), National University Health System, Singapore, Singapore; Children's National Medical Center, Washington, UNITED STATES

## Abstract

**Background:**

In 2020 the largest number of patients with coronary artery disease (CAD) will be found in Asia. Published epidemiological and clinical reports are overwhelmingly derived from western (White) cohorts and data from Asia are scant. We compared CAD severity and all-cause mortality among 4 of the world’s most populous ethnicities: Whites, Chinese, Indians and Malays.

**Methods:**

The UNIted CORoNary cohort (UNICORN) simultaneously enrolled parallel populations of consecutive patients undergoing coronary angiography or intervention for suspected CAD in the Netherlands and Singapore. Using multivariable ordinal regression, we investigated the independent association of ethnicity with CAD severity and interactions between risk factors and ethnicity on CAD severity. Also, we compared all-cause mortality among the ethnic groups using multivariable Cox regression analysis.

**Results:**

We included 1,759 White, 685 Chinese, 201 Indian and 224 Malay patients undergoing coronary angiography. We found distinct inter-ethnic differences in cardiovascular risk factors. Furthermore, the associations of gender and diabetes with severity of CAD were significantly stronger in Chinese than Whites. Chinese (OR 1.3 [1.1–1.7], p = 0.008) and Malay (OR 1.9 [1.4–2.6], p<0.001) ethnicity were independently associated with more severe CAD as compared to White ethnicity. Strikingly, when stratified for diabetes status, we found a significant association of all three Asian ethnic groups as compared to White ethnicity with more severe CAD among diabetics, but not in non-diabetics. Crude all-cause mortality did not differ, but when adjusted for covariates mortality was higher in Malays than the other ethnic groups.

**Conclusion:**

In this population of individuals undergoing coronary angiography, ethnicity is independently associated with the severity of CAD and modifies the strength of association between certain risk factors and CAD severity. Furthermore, mortality differs among ethnic groups. Our data provide insight in inter-ethnic differences in CAD risk factors, CAD severity and mortality.

## Introduction

Coronary artery disease (CAD) affects diverse populations and has become a leading global cause of morbidity and mortality.[1] The World Health Organization (WHO) reported 17 million cardiovascular deaths (30.5% of all deaths) in the year 2008 and this number is expected to rise to 23.3[2]-25[3] million by the year 2030.

While numbers of cardiovascular deaths are stabilizing or even declining in the Western world, numbers are rapidly increasing in other parts of the world.[4] This rise is most pronounced in Africa, Eastern Mediterranean regions and South East Asia; in those regions an increase of more than 10% by 2030 is predicted.[3,5]

By 2020, the highest numbers of cardiovascular deaths are expected in the Western Pacific region (~6 million) and in South-East Asia (~5 million)[5], these regions are defined by the WHO definitions[6]. Therefore, the largest part of cardiovascular deaths will be among people of Asian ethnicity. Furthermore, the probability of dying prematurely between 30 and 70 years of age from non-communicable disease, of which 48% is cardiovascular disease, is already much higher in these regions (>30%) as compared to Western Europe or North America (<20%).[3]

CAD research has been conducted predominantly among Whites, while multi-ethnic research is strongly endorsed by the American Heart Association.[7] Sizable cohort studies including Asian CAD patients are few and mostly conducted among Asian immigrants living in Western countries. These studies comparing Asians and Whites have shown some clinically important differences in risk factor burden[8,9], incidence[10] and prevalence of cardiovascular disease[11], suggesting that ethnicity influences cardiovascular risk factor burden and prevalence of cardiovascular disease. Furthermore, studies comparing regions around the globe demonstrate small, yet significant differences in the relationship between cardiovascular risk factors and cardiovascular outcomes.[12,13]

To date, there are few data directly comparing CAD risk factors and outcomes in 4 of the world’s most populous ethnic groups: Whites, Chinese, Indian and Malay (www.census.gov/popclock). We sought to identify inter-ethnic differences in CAD risk factor burden, the severity of CAD and CAD outcomes, comparing patients living in their region of origin in countries with comparable health care systems.

## Materials and Methods

### Ethics statement

The Medical Ethics Committees of both participating hospitals (Netherlands: UMC Utrecht Medical Ethics Board, Reference number: 11–183; Singapore: Domain Specific Review Boards, Office of Human Research Protection Program, Reference Number: C/10/323) approved the study and written informed consent was obtained from all patients. This study conforms to the Declaration of Helsinki.

### Study design

The UNICORN cohort (clinicaltrials.gov NCT02126150) is a multi-ethnic prospective cohort study including patients undergoing either diagnostic coronary angiography and/or percutaneous coronary intervention (PCI). The UNICORN cohort has been conducted in parallel in two countries—the Netherlands and Singapore—adhering to matched protocols. The two hospitals (UMC Utrecht in the Netherlands and National University Hospital in Singapore) are tertiary referral centers with large annual coronary angiography/PCI volumes of >3,000 and >1,500 patients, respectively. At these sites we enrolled Whites and the three largest Asian ethnic groups: Chinese, Indians and Malays.

### Study population

Consecutive patients, ≥21 years of age, undergoing coronary angiography and/or PCI for (suspected) stable or acute coronary heart disease were eligible. At inclusion, patient demographics were documented. In Singapore, trained staff recorded self-reported ethnicity as documented on state-issued identification cards using one of the following categories: Chinese, Malay, Indian and other. All Dutch patients were of self-reported White/Western-European descent.

Documentation captured cardiovascular risk factors (body mass index, hypertension, diabetes, dyslipidemia, smoking); medication use at admission (renin-angiotensin-aldosterone system (RAAS) inhibiting medication (angiotensin converting enzyme inhibitors, angiotensin II antagonists and aldosterone receptor blockers), statins, beta-blockers and platelet directed therapy (aspirin, clopidogrel, prasugrel or ticagrelor)); cardiovascular medical history, indication for coronary angiogram, coronary angiogram result and the treatment strategy for CAD.

### Angiography

Both centers used Siemens machines for coronary angiography. The Xcelera program (Philips Medical Systems) was used in both centers to store and view the recorded angiograms.

CAD severity was determined by the number of major epicardial vessels (left anterior descending coronary artery, circumflex artery and right coronary artery) with a stenosis of >50% diameter loss[14] by visual assessment of the interventional cardiologist. A significant stenosis in the left main coronary artery equated to two diseased epicardial vessels. For the current analysis CAD severity was categorized as follows: no/minor CAD, single vessel disease, double vessel disease and triple vessel disease and analyzed as an ordinal variable.

### Risk factors

Diabetes was defined as any type of diabetes (fasting glucose > 7mmol/L)[15] in the medical history or during index admission requiring medical treatment by means of oral glucose regulating medication or insulin injections (impaired glucose tolerance is not considered as diabetes in this study).

Hypertension is considered when mentioned in the patient’s medical history or when diagnosed during the index admission (systolic blood pressure > 140 mmHg or diastolic blood pressure >90 mmHg) and/or the use of one or more antihypertensiva.[16]

Dyslipidemia was defined by any dyslipidemia requiring treatment in the medical history or during index admission as recommended by the ESC/EAS[17] guidelines.

Smoking status was divided into three groups; current smoker, quit smoker (> 1 year since last smoke) and non-smoker.

Advanced renal failure was defined as any renal disease requiring treatment with oral medication or any type of renal replacement therapy in the medical history.

### All-cause mortality

The vital status of the Singaporean patients was extracted from state mortality registration and matched with individual patient data. In the Netherlands, follow-up is performed through annual patient follow-up questionnaires. When the patient did not respond, the general practitioner was contacted to obtain the patient’s vital status, which was subsequently added to the hospital registration. An extraction of the hospital registration was used for the current analysis.

### Statistical analysis

Statistical analyses were performed using the R software[18] package (version 3.0.2, Vienna, Austria). The level of statistical significance was set at α <0.05. Continuous variables (as they were normally distributed) were compared using ANOVA with Bonferroni post-hoc testing as to correct for multiple comparisons. Proportional differences were tested using a chi-square test. In order to correct for multiple testing when comparing all ethnic groups with each other, which leads to 6 tests, the level of significance was set at α 0.05/6 = 0.008 according to the Bonferroni method.

Measures of association between ethnicity and CAD severity were derived from multivariable ordinal logistic regression analyses including covariates found to be significantly associated with CAD severity (p<0.05) by univariate analyses. Covariates included age, gender, BMI, diabetes, hypertension, dyslipidemia, smoking, prior acute coronary syndrome (ACS), indication for coronary angiogram and use of anti-platelet medication, statins, beta-blocker and RAAS medication. The selected covariates were coerced in the model for all analyses (“enter model”). Stratified analyses for the independent effect of ethnicity were performed for sex, age group (≤ 62 years, >62 years, split at median age of 62 years) and diabetes status.

We tested for interactions of risk factors with ethnicity for CAD severity by adding appropriate interaction terms to the full model. The significance level for interaction was conservatively set at α<0.05.

Crude survival rates were compared by Kaplan Meier analysis with log-rank testing. Ethnicity-specific mortality rates corrected for covariates were derived from multivariable Cox regression. Patients with an “other” indication for angiography were removed from the multivariable survival analyses. Covariates were included when they were univariably significantly associated with all-cause mortality. These were: age, ethnicity, gender, indication for angiography, angiographic CAD severity, diabetes, dyslipidemia, previous ACS, statin use, platelet inhibitor use, beta blocker use and RAAS-inhibiting medication use. We compared multivariably corrected mortality rates to mortality in Malays who had the highest corrected mortality rate.

The authors had direct access to the data and take responsibility for its integrity. All authors have read and agree to the manuscript as written.

## Results

### UNICORN cohort

Enrollment commenced in September 2010 in Singapore and October 2011 in the Netherlands. By March 2014 the Netherlands had recruited 1,759 White patients and Singapore 1,110 patients (62% Chinese, 20% Malay and 18% Indians). Patient characteristics by ethnicity are shown in Table 1.

**Table 1:** Demographic and Clinical Characteristics of the UNICORN cohorts by Ethnicity.

	White	Chinese	Indian	Malay
N	1759	685	201	224
Males (%)	73.7	82.9*	83.1**	78.6
Age (years, mean ± sd)	64.9±10.9	58.6±9.8*	54.6±9.4** ^$$^	55.9±9.0^$^ ^&^
*Risk factors*				
BMI (kg/m^2^, mean ± sd)	27.0±4.5	26.1±4.7*	27.3±4.7^$$^	28.4±5.4^$^
Diabetes (%)	20.3	33.2*	51.5** ^$$^	52.2^$^ ^&^
Hypertension (%)	58.4	64.2	61.8	62.1
Dyslipidemia (%)	47.7	70.5*	77.5**	75.4^$^
*Smoking*				
Non-smoker (%)	51.0	47.4*	42.2**	42.3^$^
Quit smoker (%)	27.3	18.4	11.4	18.1
Current smoker (%)	21.7	34.2	46.4	39.6
*Medication*				
Anti platelet (%)	54.0	54.7	54.7	52.2
Statin (%)	62.2	58.4	61.2	54.5
Beta blocker (%)	57.2	42.2*	42.3**	38.8^$^
RAAS (%)	46.6	33.7*	41.8	42.9
*Medical history*				
Previous ACS (%)	29.3	16.1*	30.5^$$^	27.1^&^
Previous PCI (%)	27.9	17.4*	29.9^$$^	26.7^&^
Previous CABG (%)	10.3	6.0*	7.0	5.4
CVA/TIA (%)	9.8	5.9*	7.5	6.8
PAD (%)	11.0	2.9*	4.5**	5.4
Advanced renal failure (%)	2.1	6.0*	4.0	5.9^$^
*Indication*				
Stable CAD (%)	53.6	54.7*	47.8**	42.4^$^ ^&^
UA/NSTEMI (%)	20.1	33.1	42.3	47.3
STEMI (%)	11.3	7.6	7.5	8.0
Other (%)	14.9	4.5	2.5	2.2
*Treatment*				
Conservative (%)	34.1	47.0*	49.3**	50.0^$^
PCI (%)	59.7	44.4	46.3	39.7
CABG (%)	6.2	8.6	4.5	10.3
*Mortality*				
Follow-up time (days)	410	894	830	807
All-cause deaths (n)	56	36	5	15
Two-year mortality rate (%)	4.6	3.7	2.6	5.6

Baseline characteristics of the UNICORN cohort are displayed per ethnic group. Figures represent means ± standard deviation (sd) or percentages. Abbreviations: BMI body mass index, RAAS renin-angiotensin-aldosterone system, ACS acute coronary syndrome, PCI percutaneous coronary intervention, CABG coronary artery bypass grafting, UA unstable angina, NSTEMI non ST-elevated myocardial infarction, STEMI ST-elevated myocardial infarction. P-values for ethnic differences were calculated with a chi-square test for categorical data and one-way ANOVA for continuous, normally distributed data. The level of significance for the interethnic comparisons has been set conservatively at a p-value of 0.05/6 = 0.008 in order to correct for multiple testing.

* White vs. Chinese p<0.008.

** White vs. Indian p<0.008.

$ White vs. Malay p<0.008.

$$ Chinese vs. Indian p<0.008.

& Chinese vs. Malay p<0.008.

The proportion of males was highest in the Chinese and Indian ethnic groups (82.9% and 83.1%, respectively, p for overall difference <0.001). The Indian patients were youngest at 54.6 years compared with Malays 55.9, Chinese 58.6 and Whites who were markedly older at 64.9 years (p for overall difference <0.001).

### CAD risk factors

Diabetes was significantly more common in Malays and Indians (52.2% and 51.5%) than in Chinese and Whites (33.2% and 20.3%). Chinese had significantly lower BMI than the other ethnic groups (mean 26.1 kg/m^2^ versus >27 kg/m^2^). The prevalence of hypertension did not differ among any of the ethnic groups. Dyslipidemia was strikingly more prevalent among the three Asian ethnic groups (Chinese: 70.5%, Indian: 77.5%, Malay: 75.4%) as compared with Whites (48.1%). Higher percentages of current smokers were seen in the Asian ethnic groups, highest at 46.4% among Indians compared with 24% of White patients.

### Cardiovascular medical history

A history of previous ACS, coronary artery bypass grafting (CABG), cerebrovascular accident, transient ischemic attack or peripheral arterial disease (PAD) was less common among the Asian ethnic groups than in Whites. A history of previous ACS or PCI was significantly less common in Chinese as compared to other ethnic groups. Advanced renal failure was more common in Chinese as compared with Whites (p<0.001).

### CAD severity, presentation and treatment

**Fig 1:**
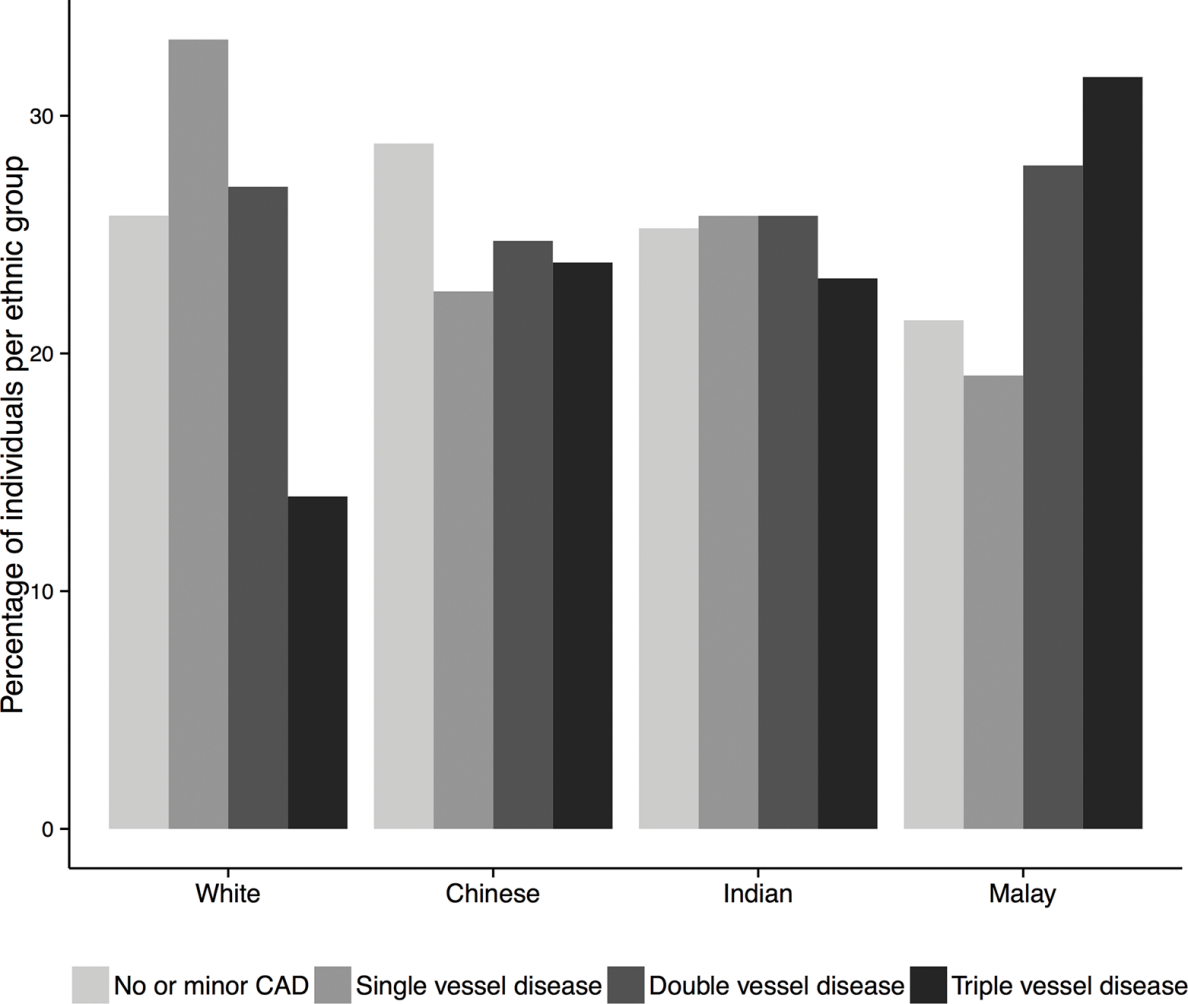
Severity of CAD by ethnicity. Bar chart depicting the distribution of CAD severity as the percentage of the total number of individuals per ethnic group. Triple vessel disease is significantly more common among Chinese, Indians and Malays than among Whites (p <0.001).

The prevalence of triple vessel disease was strikingly high in Malays (31.6%), followed by Chinese with a prevalence of 23.8% and 23.2% in Indians, as compared to Whites (14.0%, p<0.001). The distribution of the severity of CAD among the ethnic groups is depicted in Fig 1.

Approximately 11% of the White patients presented to the angiography laboratory with an ST-elevation myocardial infarction (STEMI), a significantly higher percentage as compared to Chinese (7.6%), Indians (7.5%) and Malays (8.0%). The combined category of non-ST-elevation myocardial infarction (NSTEMI) or unstable angina (UA) was more common in Chinese 33.1%, Indians 42.3% and Malays 47.3% than in Whites (20.1%).

Within the entire cohort, conservative treatment, i.e. without revascularization but encompassing risk factor control and/or anti-anginal medications, was more common in Chinese 47.0%, Indians 49.3% and Malays 50.0% than Whites 34.1%. PCI was undertaken less frequently in Chinese 44.4%, Indians 46.4% and Malays 39.7% as compared with Whites (59.7%). No inter-ethnic differences were apparent for treatment by CABG.

### Medication use

Prescription of anti-platelet medication and statins was equal across the ethnic groups (about 55%). Beta-blocker and RAAS inhibition use were most common among Whites.

Because Whites more often had a history of cardiovascular disease in the UNICORN cohort we performed a specific sub-analysis (S1 Table) of preventive drug use in participants with no history of cardiovascular disease (no acute coronary syndrome, previous PCI, CABG, cerebrovascular accidents, transient ischemic attacks or peripheral arterial disease). In this subgroup we observed that the use of anti-platelet medication and statins was equal among the ethnic groups. The use of beta-blockers and RAAS inhibiting drugs in this subgroup analysis paralleled that in the overall cohort: highest in Whites (p<0.001 for difference across all ethnic groups).

### UNICORN patients without previous CAD events

Because Whites more often had a history of CAD than the other ethnic groups, we performed a stratified analysis of inter-ethnic differences among patients with and without a history of prior CAD (no ACS, PCI or CABG). As shown in the S1 Table, we observed the same inter-ethnic distribution of risk factor burden in both strata, indicating that a past history of CAD did not modify the relationship between ethnicity and risk factor burden.

### The impact of ethnicity on the association of risk factors with CAD severity

**Fig 2:**
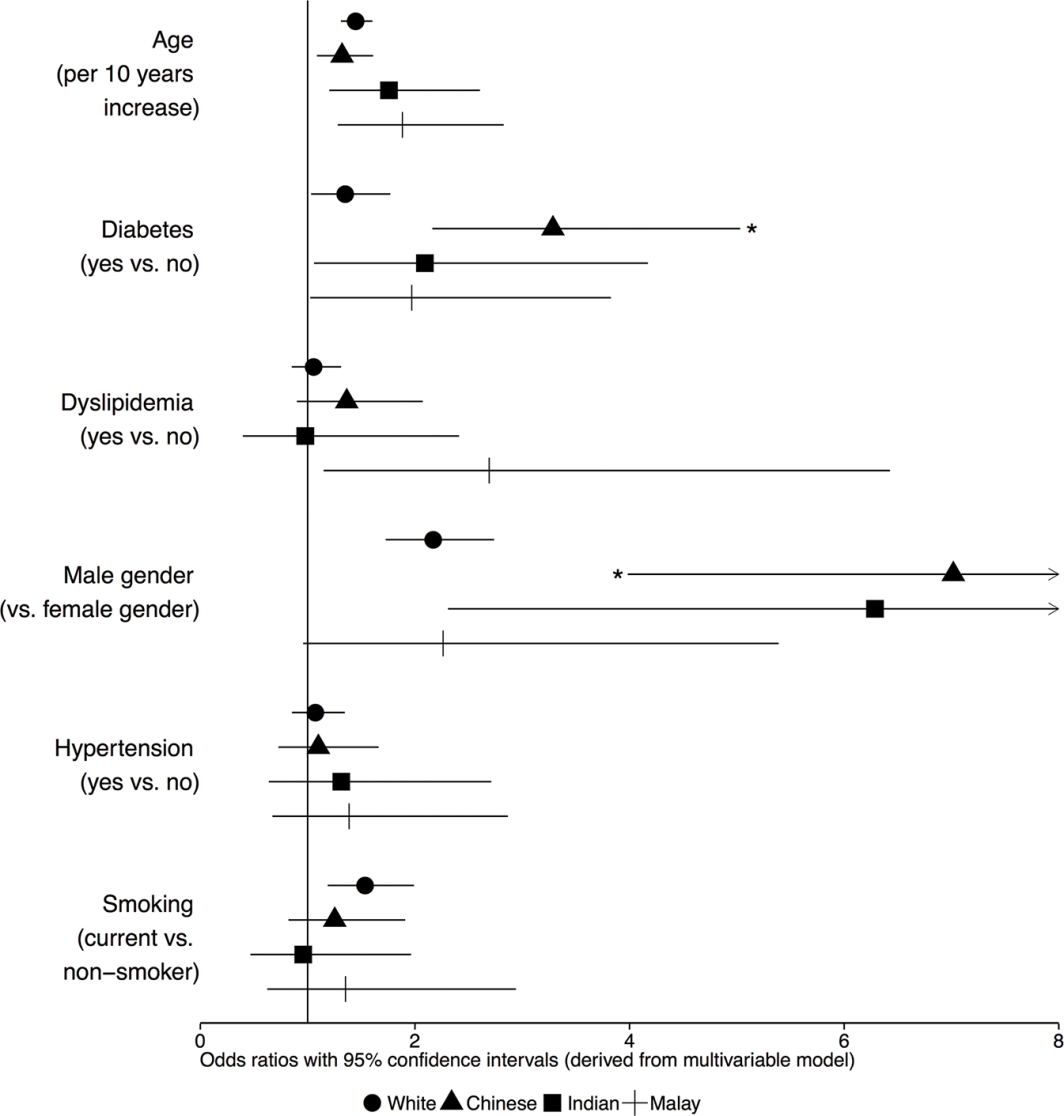
Odds ratios of risk factors for the severity of CAD by ethnicity. Odds ratios derived from multivariable ordinal regression analysis, depicting the strength of association between cardiovascular risk factors and CAD severity (categorized into no CAD, single vessel disease, double vessel disease and triple vessel disease). The point estimates and 95% confidence intervals are shown for each ethnic group. A larger odds ratio indicates a stronger association between the risk factor and CAD severity. The asterisks (*) indicate significant interactions (p<0.05) of the risk factor as compared to Whites.

The odds ratios (ORs) of specific cardiovascular risk factors for CAD severity differed between ethnic groups, as can be observed in Fig 2. The odds ratios in Fig 2 depict the ethnicity-specific odds for a given change in risk factor (for example diabetes yes or no or and increase in age of 10 years) to move up one CAD severity class (for example from single to double vessel disease, or from double to triple vessel disease).

Male gender was more strikingly associated with more severe CAD in Chinese (OR 7.0 [4.0–12.6]) than in Whites (OR 2.2 [1.7–2.7], p for interaction <0.001) and diabetes had a stronger association with more severe CAD in Chinese (OR 3.3 [2.2–5.0]) than in Whites (OR 1.4 [1.0–1.8], p for interaction 0.001).

There were no significant interactions of ethnicity with age or smoking with respect to the severity of CAD. Possible interactions of ethnicity with the relationship of dyslipidemia and hypertension to CAD severity were not tested, as these risk factors were not significantly associated with CAD severity in the multivariable model.

### Independent association of ethnicity with CAD severity

**Fig 3:**
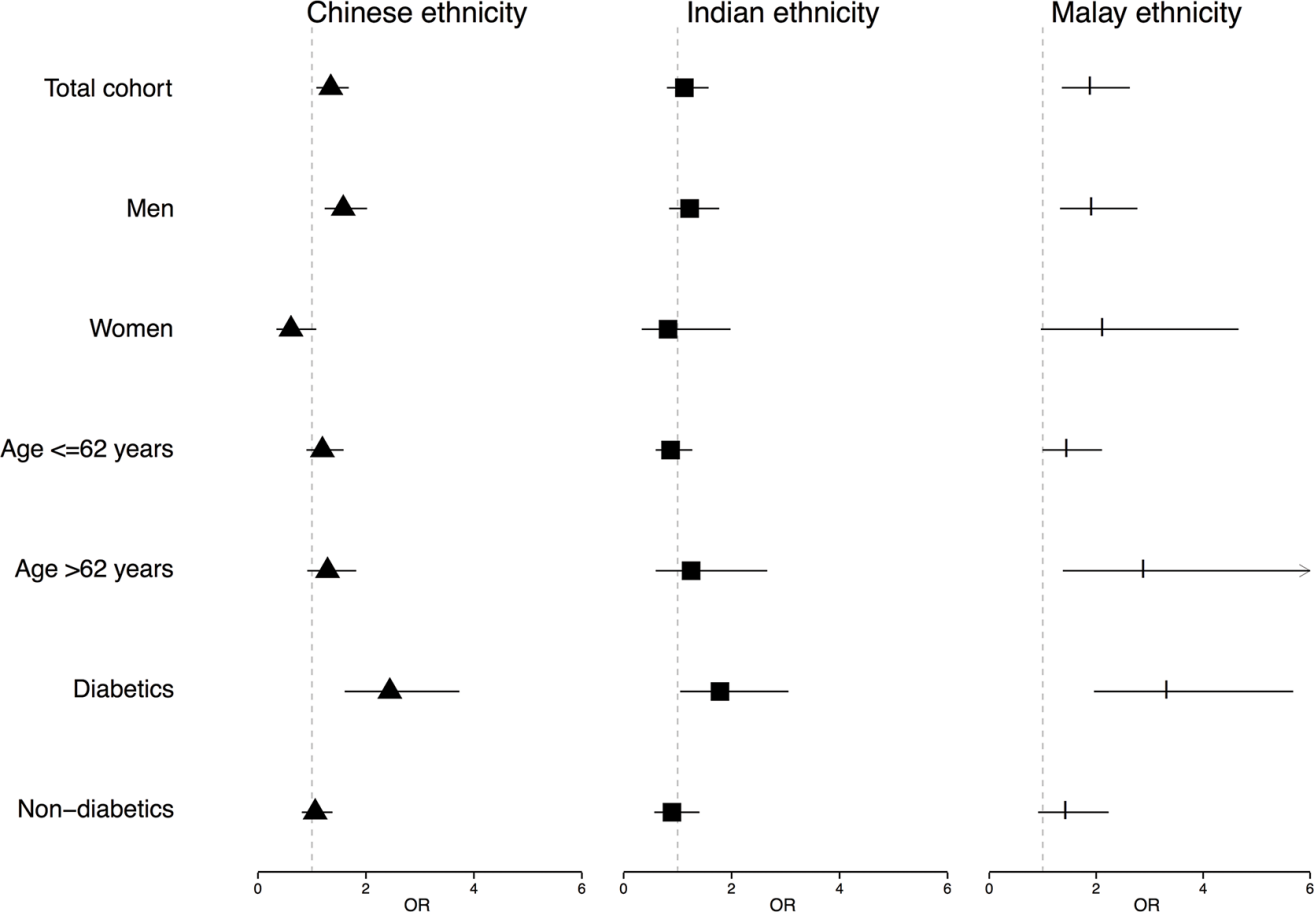
The adjusted odds ratios of Chinese, Indian and Malay ethnicity for the severity of CAD in subgroups of the UNICORN cohort. The adjusted association (odds ratios plus confidence intervals) of Chinese, Indian and Malay ethnicity as compared to White ethnicity for CAD severity, depicted for the total cohort and subgroups of the UNICORN cohort. The displayed odds ratios are derived from a multivariable model containing: age, gender, diabetes, hypertension, dyslipidemia, smoking, BMI, prior acute coronary syndrome, indication for coronary angiogram and use of anti-platelet medication, statins, beta-blocker and RAAS medication.

From a multivariable ordinal logistic regression model containing ethnicity as a covariate, we obtained ORs for Chinese, Indian and Malay ethnicity as compared to White ethnicity for the angiographic severity of CAD in the total cohort, and in specific subgroups. The results are displayed in Fig 3. Within the total cohort, ORs for Chinese and Malay ethnicity were significantly higher (1.4 [1.1–1.7] and 1.9 [1.4–2.6], respectively) using Whites as the reference group. Indicating Chinese and Malay but not Indian ethnicity, were independently associated with more severe CAD within the total cohort. This finding was largely driven by a striking interaction between ethnicity and diabetes with respect to severity of CAD. Among diabetics all Asian ethnicities were independently associated with more severe CAD as compared to White ethnicity whereas in non-diabetics this independent association of ethnicity with the severity of CAD was not observed (Fig 3).

In a sex-specific analysis, results remained similar to the total cohort among men. However, among women, Chinese ethnicity tended to be associated with less severe CAD (OR 0.6 [0.3–1.1]) as compared to White ethnicity, although not reaching statistical significance. Indian and Malay ethnicity were not significantly associated with CAD severity in women. The female subgroup, however, is small and power is markedly reduced in these analyses. This is especially the case for the Indian and Malay female subgroups, consisting of 34 and 48 women, respectively. Hence, the chance of a type II error is larger.

The ORs of severe CAD in Chinese and Indian ethnicity were comparable to Whites in those 62 years (median age) or younger compared with those over 62.

### Ethnicity and All-cause Mortality

**Fig 4:**
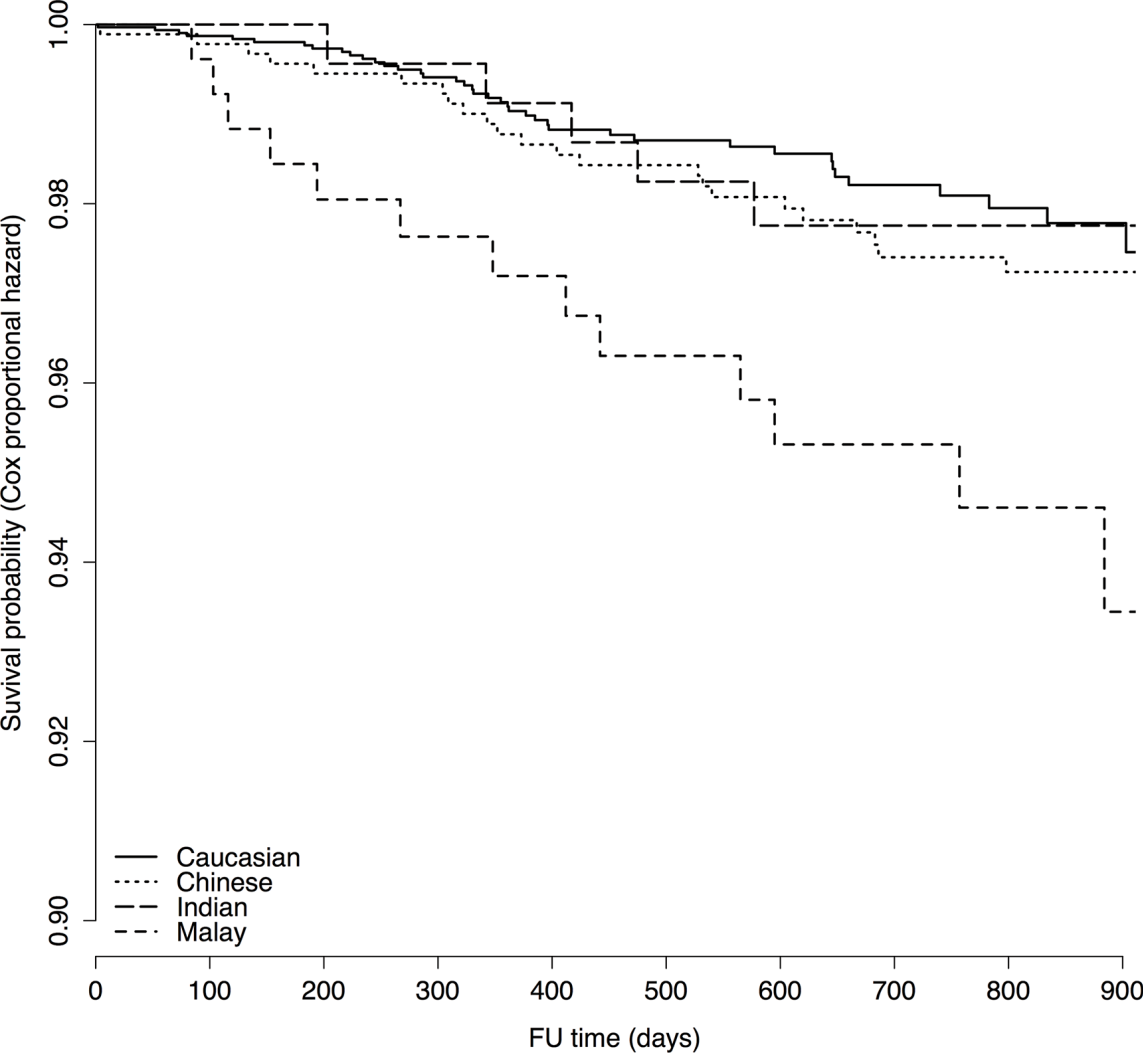
Adjusted survival probability from multivariable Cox regression analysis by ethnicity. Survival probability derived from multivariable Cox regression analysis. Ethnicity-specific curves are adjusted for: age, gender, indication for angiography, conclusion from angiography, diabetes, dyslipidemia, previous ACS, statin use, platelet inhibitor use, beta blocker use and RAAS-inhibiting medication use. White, Chinese and Indian ethnicity were significantly associated with a better survival as compared to Malay ethnicity (Whites: HR 0.4 [0.2–0.8], p = 0.009, Chinese: HR 0.5 [0.3–0.98], p = 0.044, Indians HR 0.4 [0.1–0.98], p = 0.046).

Crude all-cause mortality rates (from Kaplan Meier analysis) showed lowest survival probability among Whites, and highest in Indians (Table 1, log-rank test for difference across the ethnic groups p = 0.17). On correction for covariates by Cox regression analysis to assess the independent effect of ethnicity on all-cause mortality, survival in Malays fell below that in Chinese, Indians and Whites (Fig 4). White, Chinese and Indian ethnicity were significantly associated with a better survival as compared to Malay ethnicity (Whites: HR 0.4 [0.2–0.8], p = 0.009, Chinese: HR 0.5 [0.3–0.98], p = 0.044, Indians HR 0.4 [0.1–0.98], p = 0.046).

## Discussion

In the UNICORN study, we compared four of the most populous ethnic groups in the world, living in two countries that are comparable in terms of development: Singapore and the Netherlands. Both countries are ranked within the top 20 on the human development index[19] and have comparable health care systems.[20]

We defined ethnic differences in cardiovascular risk factors, the severity of CAD and all-cause mortality in patients undergoing coronary angiography. Chinese and Malay ethnicity were independently associated with more severe CAD compared to White ethnicity. This finding was largely driven by a striking interaction between ethnicity and diabetes with respect to CAD severity. Ethnicity also interacted with male gender, modifying its association with CAD severity.

Mortality was highest among Malays, this difference in all-cause mortality after coronary angiography persisted after adjustment for baseline differences.

### UNICORN characteristics

Our results show clear ethnic differences in age at presentation. Previous studies have shown that Indians (South Asians) tend to incur CAD at a younger age, indicating a higher atherosclerotic burden earlier in life.[10,21–23] With respect to other Asian ethnic groups; in the e-HEALING[24] coronary stent registry of Asians from Singapore, Hong Kong and Malaysia, the mean age of Whites (from Western Europe) was 65.9, whilst the mean age of Asian registrants was 57.4 years, very much in line with our cohort. These prior reports highlighted key differences between White and Asian patients with CAD but Asians were typically classified as a single ethnic group. In the current study, we clearly demonstrate that significant and clinically relevant differences in age at presentation also exist among the Asian ethnic groups. Our data underscore the importance of delineating specific Asian ethnicities to avoid missing key inter-ethnic differences.

### Risk factor burden

The existing literature, mainly derived from populations living within western communities, has mainly focused on the risk factor burden of South Asians (often residents of the UK[25] or US[9]) as compared to Whites, and shows a higher burden in South Asians, which we also observe. Chinese have been found to display a slightly more benign risk factor pattern compared to Whites in population-based studies.[26,27] However, in Chinese individuals with overt cardiovascular disease, a higher risk factor burden has been reported, corresponding to our findings.[28,29] Risk factors in Malays as compared to Whites have been less well documented in the literature, but striking differences were encountered in this study.

### CAD severity

In our cohort we find a higher prevalence of angiographic triple vessel disease in Chinese, Indians and Malays as compared to Whites. However, differences remained after adjustment for baseline differences in risk factors. This indicates that the differences in risk factor burden only partly explain the more severe CAD phenotype that is observed in Chinese, Indians and Malays. Apparently, ethnicity conveys an important independent (biological or life-style mediated) component that is not fully captured by the general patient characteristics or risk factor burden and warrants more detailed research.

Importantly, these differences appeared to be largely driven by diabetes with risk of severe CAD clearly enhanced compared with non-diabetics to a greater degree in all three Asian ethnicities than the additional risk conferred by diabetes in Whites.

Besides the independent association of ethnicity with CAD severity, we also find significant interactions of ethnicity with cardiovascular risk factors. It thus appears that ethnicity also modifies the effect of certain risk factors on CAD severity. This modifying effect has been rarely studied, although stronger associations of cholesterol levels and diabetes with carotid intima-media thickness have been reported by Chow et al.[30] Their findings might indicate that the vascular wall of South Asians is more susceptible to glycemic and lipidemic disturbances than of Whites.

The mechanisms underlying both the independent impact of ethnicity, as well as the modifying effect of ethnicity remain to be elucidated.

### Mortality

South Asians have a higher incidence of CAD, but lower[29,31,32] or comparable[33,34] (coronary) mortality rates as compared to Whites. In Chinese survival similar[28] or better[29] than in Whites has been reported. Our results largely concur with existing literature, showing similar corrected survival probabilities for Indians, Chinese and Whites. However, to our knowledge, for Malays no comparison with Whites has been previously published. Although, a comparison among the Asian ethnic groups in Singapore, showed higher all-cause mortality in Malays as compared to Chinese and Indians among myocardial infarction patients.[35] Accordingly, we found survival in Malays to be lower than in the other ethnic groups in both crude and corrected analyses, indicating that the high burden of risk factors and the more severe CAD are accompanied by higher mortality rates in Malays.

### Implications and future directions

The most striking interaction we observed between ethnicity and risk factors on CAD severity was observed for diabetes. Specific focus might be granted to stricter glycemic control among Chinese in whom diabetes has the biggest impact on CAD severity. Earlier CAD screening might be appropriate among Chinese and Malay men, as Chinese and Malay ethnicity are independent predictors of more severe CAD in men, but not in women.

Malays, with the heaviest burden of risk factors suffered the poorest survival. The proportion of conservative treatment was highest and use of preventive medications lowest among Malays whilst they carried the most severe CAD. More vigilant surveillance and more aggressive pharmacological and interventional treatment of CAD might be explored in this ethnic group.

In order to elucidate inter-ethnic differences studies assessing dietary and life-style habits, as well as multi-ethnic biobanking initiatives offer valuable perspectives.[36] Cultural and socioeconomic factors might influence risk of CAD and CAD severity among the ethnic groups. A careful approach in unraveling ethnicity-dependent patterns of diet, physical activity and other life-style habits might elucidate a substantial part of the risk that is conveyed by ethnicity. Although not available in our dataset, income levels are known to differ among the four examined countries and ethnic groups, being higher in Singapore than in the Netherlands: with a per capita GDP of US$ 55,182 and US$ 50,793 in the Netherlands (data for 2010–2014 from data.worldbank.org). Within Singapore the median monthly income differs markedly among the ethnic groups: S$5,100 for Chinese, S$3,844 for Malays and S$5,370 for Indians.[37] The median monthly income in the Netherlands is estimated at €2,391[38] roughly corresponding to S$3571. While substantial differences are visible in the income levels among the ethnic groups, it might not necessarily mean that income affects CAD risk and severity in similar ways among the ethic groups. Per ethnic group, physical activity, dietary and health care utilizing behavior of poorer or richer individuals might be different. These habits must be elucidated in an ethnicity-specific manner in order to indicate possible targets for cardiovascular health improvement, which might best be done through qualitative research.

Additionally, biomarkers related to CAD have been found to differ between Whites and certain Asian ethnic groups on a general population level.[39] Biomarker differences, yet to be revealed among the ethnic groups, might guide us to biological pathways involved in the accelerated and more severe CAD observed in Indians and Malays.

### Limitations

The largest limitation to our study is that we were unable to correct for dietary, life-style and socioeconomic factors[40] as possible confounders. It is possible that these factors would influence our results, however we expect that by correcting for many other CAD risk factors the effect of life style and socioeconomic status has been covered for a large part.[41]

Although Singapore and the Netherlands are fairly comparable[19,20] when it comes to development status and health care systems, we could not correct for possible differences in these factors. It is possible that differences in, for example, the referral habits of primary care and secondary care physicians between Singapore and the Netherlands have an impact on the ethnic differences we observe. However, if this would be the case one would not expect many differences within the Asian ethnic groups or between the sexes (within one ethnic group), which we do find. Also, clinical decision-making and patient preferences for invasive coronary investigations might differ between the countries and among the ethnic groups.[42]

Our results are exclusively applicable to patients who have undergone coronary angiography in a tertiary care center: the most symptomatic group of CAD patients. Caution should be exercised when extrapolating our results to the entire CAD population. Additional studies are needed to examine to which extent our results are applicable to other CAD populations.

Due to a limitation in statistical power no sex-specific or other stratified analyses could be performed on the mortality data.

## Conclusion

Striking differences were found between Whites, Chinese, Indians and Malays undergoing coronary angiography for suspected CAD. Ethnicity is independently associated with the severity of CAD and all-cause mortality after coronary angiography. Notably, Chinese ethnicity alters the strength of association between established cardiovascular risk factors (diabetes and male gender) and CAD severity.

Our data gain insight in the characteristics of coronary artery disease among the ethnic groups and suggest that CAD management should account for the differential impact of risk factors on CAD severity among different ethnicities. Similar studies in the other cardiovascular disease populations would be useful.

## Supporting Information

S1 Table Baseline characteristics of the UNICORN participants with no history of CAD events. Baseline characteristics of the UNICORN participants with no history of CAD events with p-values for the differences among the ethnic groups (derived from chi-square and ANOVA tests).
(PDF)
