# Supplementary material for: Ethnicity Modifies Associations between Cardiovascular Risk Factors and Disease Severity in Parallel Dutch and Singapore Coronary Cohorts
Source: PLoS One. 2015 Jul 6;10(7):e0132278. doi: 10.1371/journal.pone.0132278 (PMC4492665; doi:10.1371/journal.pone.0132278)
Supplement: S1 Table — Baseline characteristics of the UNICORN participants with no history of CAD events with p-values for the differences among the ethnic groups (derived from chi-square and ANOVA tests). (PDF) [file pone.0132278.s001.pdf]

Supplemental Table. Baseline characteristics of the UNICORN participants with no history of CAD events

	**White**	**Chinese**	**Indian**	**Malay**	*P-value difference*
N	914	516	121	142	
Males (%)	69.1	81.6	78.5	76.1	<0.001
Age (years, mean (sd))	63.5 (11.4)	57.9 (9.8)	53.0 (9.5)	54.4 (8.7)	<0.001
Risk factors					
BMI (kg/m^2, mean (sd))	26.6 (4.5)	26.1 (4.6)	27.7 (4.9)	28.7 (5.7)	<0.001
Diabetes (%)	15.6	28.9	48.3	48.6	<0.001
Hypertension (%)	53.6	61.2	53.8	57	0.048
Dyslipidemia (%)	38.0	67.0	67.5	68.3	<0.001
Smoking					
Current smoker (%)	52.6	50.1	43.6	47.3	<0.001
Quit smoker (%)	24.1	14.3	8.9	15.5	
Non-smoker (%)	23.3	35.5	47.5	37.3	
Medication					
Anti platelet (%)	43.7	43.6	34.7	33.8	0.044
Statin (%)	46.2	48.8	43.8	40.1	0.283
Beta blocker (%)	42.9	30.0	22.3	26.8	<0.001
RAAS (%)	39.9	24.6	25.6	30.3	<0.001
Medical history					
CVA/TIA (%)	8.6	5.0	5.8	5.6	0.066
PAD (%)	8.0	2.3	1.7	3.5	<0.001
Advanced renal failure (%)	1.5	3.7	4.1	4.2	0.028
Indication					
Stable (%)	44.2	55.2	51.2	41.5	<0.001
UA/NSTEMI (%)	21.9	30.2	33.9	45.1	
STEMI (%)	17.4	9.3	11.6	10.6	
Other (%)	16.5	5.2	3.3	2.8	
Angiographic finding					
No CAD (%)	30.8	32.6	29.8	27.5	<0.001
1-Vessel Disease (%)	30.1	23.6	28.1	17.6	
2-Vessel Disease (%)	24.0	23.1	24.8	24.6	
3-Vessel Disease (%)	15.1	20.7	17.4	30.3	
Treatment					
Conservative (%)	35.2	46.9	50.4	51.4	<0.001
PCI (%)	58.0	44.0	45.5	38	
CABG (%)	6.9	9.1	4.1	10.6	

Supplemental table. Baseline characteristics of the UNICORN participants with no history of CAD events with p-values for the differences among the ethnic groups (derived from chi-square and ANOVA tests).
